# Estimation of Specific Cutting Energy in an S235 Alloy for Multi-Directional Ultrasonic Vibration-Assisted Machining Using the Finite Element Method

**DOI:** 10.3390/ma13030567

**Published:** 2020-01-24

**Authors:** Luis C. Flórez García, Hernán A. González Rojas, Antonio J. Sánchez Egea

**Affiliations:** 1 Department of Mechanical Engineering, Universidad Tecnológica de Pereira, Risaralda 660003, Colombia; louis@utp.edu.co; 2 Department of Mechanical Engineering (EPSEVG), Universitat Politécnica de Catalunya, 08800 Barcelona, Spain; hernan.gonzalez@upc.edu; 3 Department of Mechanical Engineering (EEBE), Universitat Politècnica de Catalunya, 08019 Barcelona, Spain

**Keywords:** vibration-assisted turning, machinability, specific cutting energy, finite element method, elliptical motion, duty cycle

## Abstract

The objective of this work is to analyze the influence of the vibration-assisted turning process on the machinability of S235 carbon steel. During the experiments using this vibrational machining process, the vibrational amplitude and frequency of the cutting tool were adjusted to drive the tool tip in an elliptical or linear motion in the feed direction. Furthermore, a finite element analysis was deployed to investigate the mechanical response for different vibration-assisted cutting conditions. The results show how the specific cutting energy and the material’s machinability behave when using different operational cutting parameters, such as vibration frequency and tool tip motion in the *x*-axis, *y*-axis, and elliptical (*x*-*y* plane) motion. Then, the specific cutting energy and material’s machinability are compared with a conventional turning process, which helps to validate the finite element method (FEM) for the vibration-assisted process. As a result of the operating parameters used, the vibration-assisted machining process leads to a machinability improvement of up to 18% in S235 carbon steel. In particular, higher vibration frequencies were shown to increase the material’s machinability due to the specific cutting energy decrease. Therefore, the finite element method can be used to predict the vibration-assisted cutting and the specific cutting energy, based on predefined cutting parameters.

## 1. Introduction

Reliability, flexibility, visual computing, optimization, and integration into the industrial scenario are manufacturing characteristics to be addressed in the Industry 4.0 philosophy and the circular economy [1]. In this philosophy, the use of virtual models based on finite element methods (FEM) is playing a significant role in obtaining a numerical approach to complex problems [2]. In particular, for the last two decades, the scientific community broadly used FEM to model different manufacturing processes, such as chip removal and plastic deformation [3]. Focusing on the material removal processes, FEM analysis lets us locally investigate the transition between the elastic and plastic deformation stages, which is difficult to determine during experimental trials [4]. Accordingly, FEM modeling helps us to understand the complex phenomena that occur in the cutting zone [5]. For example, during a stationary orthogonal machining process, FEM helps to determine the importance of chip morphology, cutting zone temperature [6], cutting stresses [7], tool behavior, burr formation [8], and the cutting force [9]. The cutting force is one of the most studied parameters because it is easy to validate experimentally [10]. FEM was also used to study vibration-assisted machining (VAM), where the cutting tool moves in one or two axes. VAM deploys an oscillating cutting progression and, subsequently, an oscillating cutting force. In this regard, empirical, predictive, and computational modeling are addressed [11]. In general, empirical models are widely found when modeling VAM, independently of whether the cutting tool moves in one direction (VAM-1D) [12] or two directions (VAM-2D) [13]. Also, the combination and comparison of empirical and predictive models help to reduce error in the force prediction when VAM-2D is analyzed [14]. Thus, the cutting forces are reduced, on average, when using VAM-2D [15], and the tool life is enhanced due to reduced wear [16]. Moreover, the oscillating cutting progression has an essential impact on the surface properties of the material cut. In particular, VAM-2D improves the properties of the surface finish compared to conventional machining and VAM-1D [17]. These surface property enhancements using VAM-2D were experimentally found in machining with diamond tools [18], micro tools for wire cutting [19], conventional tools [14], and micro- and nano-dimensional machining [13]. The use of FEM to model VAM is mainly deployed assuming an orthogonal cutting process in which the cutting tool moves either in one or two directions. For example, Shamoto and Suzuki [18] investigated the cutting force and the contact length during an elliptical movement (movement in two dimensions), and Dali et al. [20] modeled, using FEM, an orthogonal VAM-1D cut with tool vibration parallel to the feed rate. Furthermore, Zhu et al. [21] studied, theoretically and experimentally, the cutting forces and surface characteristics when using VAM-2D with an elliptical movement to cut a brittle ceramic. They compared the force values obtained, between vibration and stationary machining, and showed that the averaged value of the cutting forces was reduced up to 90% for a VAM-2D.

The literature focused on studying the cutting forces or the surface properties, but few studies investigated the specific cutting energy (SCE) via numerical simulation [22]. The SCE lets us define the material machinability [23], which is usually between 15% and 70% of the total energy consumed [24]. Consequently, the efficiency of the process can be improved by reducing the energy required to remove material [25], where SCE is a key indicator of the energy consumed by cutting [23]. The energy consumption in metal cutting processes is a function of the cutting speed, feed rate, depth of cut, and the type of material to be cut [26]. Pawade et al. [27] reported that the SCE has an important effect on chip formation, cutting force, tool wear, and surface properties. The SCE increases if the feed rate and cutting depth are reduced when turning low/medium-carbon steels [28]. Therefore, SCE is a critical parameter when characterizing the experimental chip removal process. Thus, great efforts still need to be made to understand and to estimate the SCE in VAM (1D or 2D), due to the non-linear behavior of the material cut, the cutting geometry, machining mechanism, material failure, and temperature in the cutting zone, as well as other aspects [3]. Accordingly, the present work focuses on investigating, using FEM, the influence of the multi-directional VAM process on the cutting forces and the specific cutting energy. To do this, an orthogonal vibration cutting process is deployed where the cutting tool moves in one (VAM-1D) or two (VAM-2D) directions, to determine the SCE and machinability of the S235 steel alloy. Finally, SCE was validated with the experimental data obtained from conventional turning.

## 2. Model of Vibration-Assisted Machining

A VAM process is characterized and modeled in this section. Firstly, the cutting process is modeled as a stationary orthogonal cutting, where the cutting tool moves in one or two axes in the cutting plane. Then, the behavior of the SCE is modeled in vibrating and non-vibrating processes. Non-vibrating cutting is used to validate the FEM model, since there are many experimental reports of the behavior of SCE in different cutting processes, such as turning [28], drilling [29], and sawing [30]. Once the model is adjusted and validated for a non-vibration mode, vibration machining is deployed with FEM in one direction (VAM-1D) and two directions (VAM-2D).

### 2.1. Cutting Conditions for VAM-1D and VAM-2D

**Figure 1:**
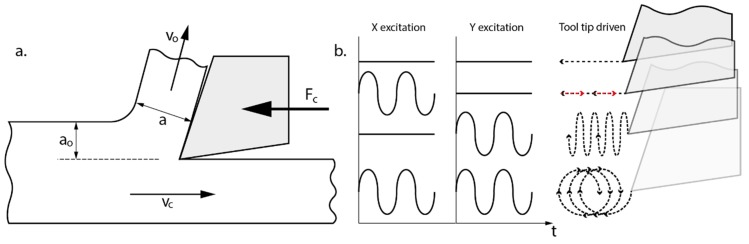
(**a**) Cutting parameters in an orthogonal cutting process; (**b**) relative movements of the cutting tool for the different studied vibration modes.

The operational parameters for a conventional turning process are cutting speed, cutting feed, and cutting depth. In orthogonal cutting, cutting speed is the horizontal speed (*Vc*), cutting feed (*f*) is the undeformed chip thickness (*a_0_*) per revolution, and cutting depth is the magnitude perpendicular to the cutting plane (see Figure 1a). Combinations of movements performed by a multi-directional vibration tool are shown in Figure 1b. A carbide tool DIN4980-ISO6 P20 fitted with diamond ground carbide, inserted with a rake angle of 6° and a nose radius of 0.2 mm and held by a standard tool holder, was used to machine the metallic bars. Relative movements of the cutting tool during VAM are (a) without vibration movement, (b) vibration in the horizontal axis VAM-1Dx, (c) vibration in the vertical axis VAM-1Dy, and (d) vibration in both axes VAM-2D. Then, the average cutting force (*F_C_*) for all these vibration modes was analyzed and compared, by changing the vibration frequency (*f_0_*).

The equations governing the vibration motion of the tool on the *x*- and *y*-axes are defined in Equation (1).

(1) $$\begin{array}{c} \mathrm{VAM}-1\mathrm{Dx}\{\begin{array}{c} x\left(t\right)=Ax\cdot sin\left(2\cdot \pi \cdot f_{0}\cdot t\right)+V_{c}\cdot t\\ y\left(t\right)=0 \end{array},\\ \mathrm{VAM}-1\mathrm{Dy}\{\begin{array}{c} x\left(t\right)=V_{c}\cdot t\\ y\left(t\right)=Ay\cdot sin\left(2\cdot \pi \cdot f_{0}\cdot t\right) \end{array}, \end{array}$$

where *Ax* and *Ay* are the amplitudes of vibration on each axis, and f*_0_* is the vibration frequency of the cutting tool. VAM-2D presents an elliptical toolpath with amplitudes *Ax* and *Ay* in the *x*- and *y*-direction, respectively. Although that VAM has the same vibration frequency in both directions, a frequency mismatch (β) is present in the horizontal direction, as shown in Equation (2).

(2) $$\mathrm{VAM}-2D\{\begin{array}{c} x\left(t\right)=Ax\cdot sin\left(2\cdot \pi \cdot f_{0}\cdot t+\beta \right)+V_{c}\cdot t\\ y\left(t\right)=Ay\cdot sin\left(2\cdot \pi \cdot f_{0}\cdot t\right) \end{array}$$

Velocity and acceleration of the tool movement are acquired by derivation of Equations (1) and (2) with respect to time for each vibration mode. Then, the range of values of vibration frequency and amplitudes (Ax and Ay) are used in the FEM simulation to characterize the VAM modes listed in Table 1.

### 2.2. Numerical Approach to Orthogonal Cutting

**Figure 2:**
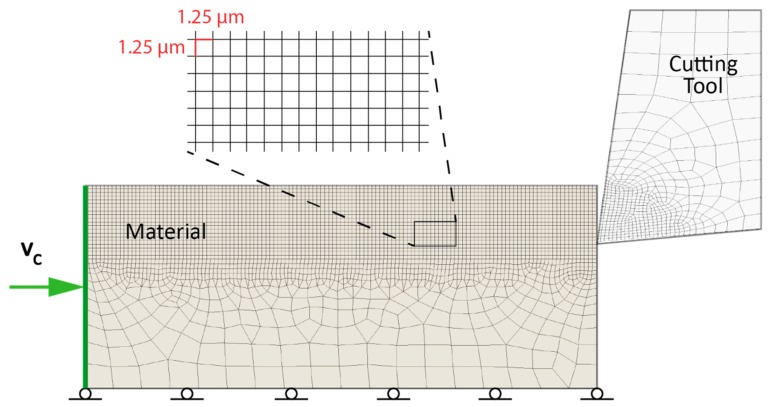
Initial and boundary conditions of an orthogonal cutting modelled by finite element method (FEM).

The orthogonal cutting is modeled by two bodies which represent the geometric limits of the cutting tool and the material to be cut (see Figure 2). The geometric intersection of both bodies produces the chip removal process. In the stationary mode, the material moves from left to right, while the cutting tool does not have any relative movement. Accordingly, boundary conditions for the material are the cutting velocity (*V_C_*) applied on the left side and frictionless support at the bottom. Cutting velocity is assumed constant as an initial condition in the material to be cut; the rest of the conditions and material properties are defined in Table 2. 

Partial differential equations associated with an explicit dynamic analysis were used to express the conservation of mass, momentum, and energy in Lagrangian coordinates. Subsequently, these equations, together with a constitutive model and the initial and boundary conditions, allowed the mechanical response to be fully defined [31]. Then, the FEM model was solved by using ANSYS (version 19.2, Ansys Inc., Canonsburg, PA, USA) with explicit dynamics equation, using Lagrangian formulation in the plastic deformation zone. A mesh of 75,556 first-order quadrangular elements and 76,385 nodes was used, where the approximate length of the element was 0.00125 mm, and the integration time step was 1 × 10^−12^ s. Finally, the plastic behavior of the S235 carbon steel was defined by the constitutive equation of Johnson–Cook.

(3) $$\sigma =\left(A+B\varepsilon^{n}\right)\left(1+C\cdot ln\frac{\overset{´}{\varepsilon }}{\overset{´}{\varepsilon_{0}}}\right)\left(1-{\left(\frac{T-T_{room}}{T_{melt}-T_{room}}\right)}^{m}\right)$$

The constants A, B, and C of the Johnson-Cook equation to define the behavior of S235 carbon steel are shown in Table 3 and obtained from the reported works of Martinez et al. [32] and Verleysmen et al. [33].

The numerical scheme for solving the cutting problem is an explicit algorithm for nonlinear dynamics with FEM [31]. This scheme was used to solve the problem of cutting without vibration, as well as the problem of cutting with vibration. The FEM study of a cut without vibration permitted the validation of the correct function of the algorithm in a known problem. The extensive experimental information on the problem allowed us to validate the modeling. We assumed the results of the simulation of cutting without vibration to be correct. Then, the algorithm was used to solve the problem of cutting with vibration, for which there is little experimental information allowing its validation.

## 3. Results and Discussion

The results and discussion of the stationary and multi-directional (1D and 2D) vibrational orthogonal cutting modes are presented in this section, in order to compare and analyze the cutting forces, the stress-strain distribution in the shear zone, and the SCE.

### 3.1. Stationary Orthogonal Cutting

**Figure 3:**
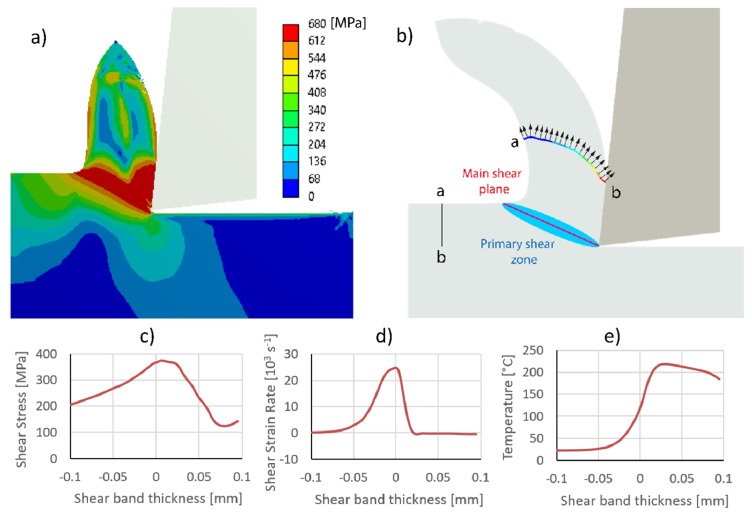
A numerical model of the stationary orthogonal cutting to study: (**a**) von Mises stress, (**b**) primary shear zone, (**c**) shear stress distribution, (**d**) rate of shear deformation, and (**e**) temperature.

Figure 3a shows the von Mises stress distribution at the chip formation. The maximum values are found in a band around the primary cutting zone, a band that is usually defined by the angle of the cutting plane, of about 22.5° [34]. Assuming that the shear band thickness is around the primary deformation zone [35], the values for maximum stress, shear strain rate, and temperature in that shear band were analyzed (see Figure 3b). This band is considered to be in one dimension, and the magnitudes are a function of a coordinate in the direction perpendicular to the main shear plane. Then, Figure 3c exhibits the behavior of shear stress as a function of shear band thickness. The maximum shear stress is around position 0 of the shear band, which corresponds to the hypothetical cutting plane. In Figure 3d, the shear rate increases up to a maximum value above the cutting plane, and then rapidly decreases to zero. This maximum shear strain rate value obtained by FEM is of the same order of magnitude as obtained by Tounsi et al. [35]. The temperature distribution observed in Figure 3e also increases from room temperature up to a maximum value above the cutting plane, and then remains relatively constant [36]. The shear strain rate allows us to define the start and end of the shear band thickness. Also, the shear strain rate distribution is not symmetrical to the cutting plane and is a function of the proportion of the main shear zone (*α*) [35]. In the numerical simulation, the main shear zone and the shear band thickness are estimated from the area under the curve of the shear strain rate, where the left and right boundaries are defined to cover the 99% of the total area. The difference between the right and left boundaries defines the shear band thickness. Komanduri et al. [36] stated that the shear band thickness could be approximated at one-half of the uncut chip thickness. However, the quotient between the uncut chip thickness and the shear band thickness is considered here. The numerical estimation obtained from the simulation shows that, with a probability of 95%, the quotient is within the interval of 1.64-1.83, and the main shear zone is within 0.78–0.82. Similar ranges are also found in experiments performed by Tounsi et al. [35] and Binglin et al. [37]. FEM uses sudden failure models [38] or damage evolution models [39]. The fracture model for this study was a constant fracture strain, which generates an approximate behavior of the material.

The horizontal (Fx) and vertical (Fy) cutting forces estimated at the tool were 98 N and −4 N, respectively. Then, SCE was determined with Equation (4), which relates to the cutting power (*N_C_*) and the material removal rate (*Q_W_*). The cutting power is defined as the product of the cutting force (*F_C_*) by the cutting speed (*V_C_*). The material removal rate is defined by the product of the output speed of the chip (*V_O_*) and output chip section (*A_O_*). This section is determined by following the nodes within the a-b line (see Figure 3b). Firstly, the a-b line is marked in the non-deformed zone, and then these nodes are identified after the plastic deformation of the cutting process. Once the a-b line is deformed, the average output speed of the chip is estimated using Equation (5).

(4) $$SCE=\frac{F_{c}\cdot V_{c}}{V_{0}\cdot A_{0}},$$

(5) $$V_{o}=\frac{1}{\sum L_{i}}\sum V_{i}\cdot L_{i},$$

where $L_{i}$ is the length of each mesh element, and $V_{i}$ is the normal component of the speed for each element.

Table 4 shows the compression ratio, shear plane angle, and SCE found using the above-presented FEM and the experimental values reported by Hameed et al. [40] in S235 carbon steel. They experimentally analyzed these parameters using different values of cutting speed, feed rate, and depth of the cut. The prediction of the chip compression ratio was found to be similar to the experimental magnitude. However, larger error prediction found at higher cutting speed, due to the estimation of plastic behavior at high shear strain rate, is a phenomenon difficult to capture with an FEM model. The shear plane angle density is not accurately represented by the FEM simulation, likely due to the fact that the primary deformation zone is defined by a cutting plane, which is a coarse assumption. In particular, Figure 3a shows a deformation region where the highest shear stresses are exhibited and, consequently, there is not a single deformation plane. Finally, the estimation of SCE is within one order of magnitude of the experimental data. For higher cutting feeds or higher cutting speeds, lower numerical and experimental SCE values are found. The FEM simulation of a stationary orthogonal cutting shows similar results to those experimentally obtained in a previous study [41], which validates the model used in the present work. Accordingly, as the stationary mode was validated, the next section deals with the problem of VAM mode in different directions.

### 3.2. Multi-Directional Vibration-Assisted Orthogonal Cutting

**Figure 4:**
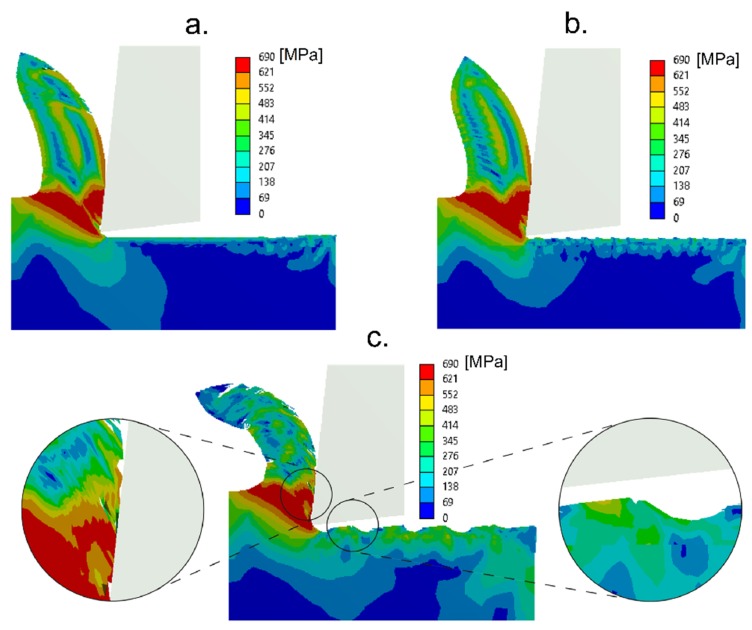
Distribution of von Misses stresses along with the chip formation and material surface for VAM-1Dx (**a**), VAM-2D (**b**), and VAM-1Dy (**c**).

FEM simulations were performed to analyze the vibration-assisted cutting when four vibration frequencies of 10, 20, 30, and 40 kHz were used. Figure 4 shows the chip formation and the von Mises stress in the primary cutting zone for VAM-1Dx, VAM-2D, and VAM-1Dy. The von Mises stress distribution in the chip formation in horizontal and elliptical vibration modes presented a similar magnitude. For the vertical vibration mode, the stress distribution changed, and micro-cracks appeared on the chip face, which was in contact with the rake face of the cutting tool. There were also irregularities in the material surface which was in contact with the flank face of the cutting tool. These irregularities were probably due to collisions of the tool with the surface of the material, due to the lack of continuous contact between the tool and material. The collision with the surface was a scenario of great plastic deformations that occurred for time spans of 1 × 10^−12^ s, which is a situation difficult to capture by FEM simulation. For this reason, the principle of energy conservation was not ensured in VAM-1Dy. The error prediction rate was larger than 10%, which is the limit to use of the numerical approach.

**Figure 5:**
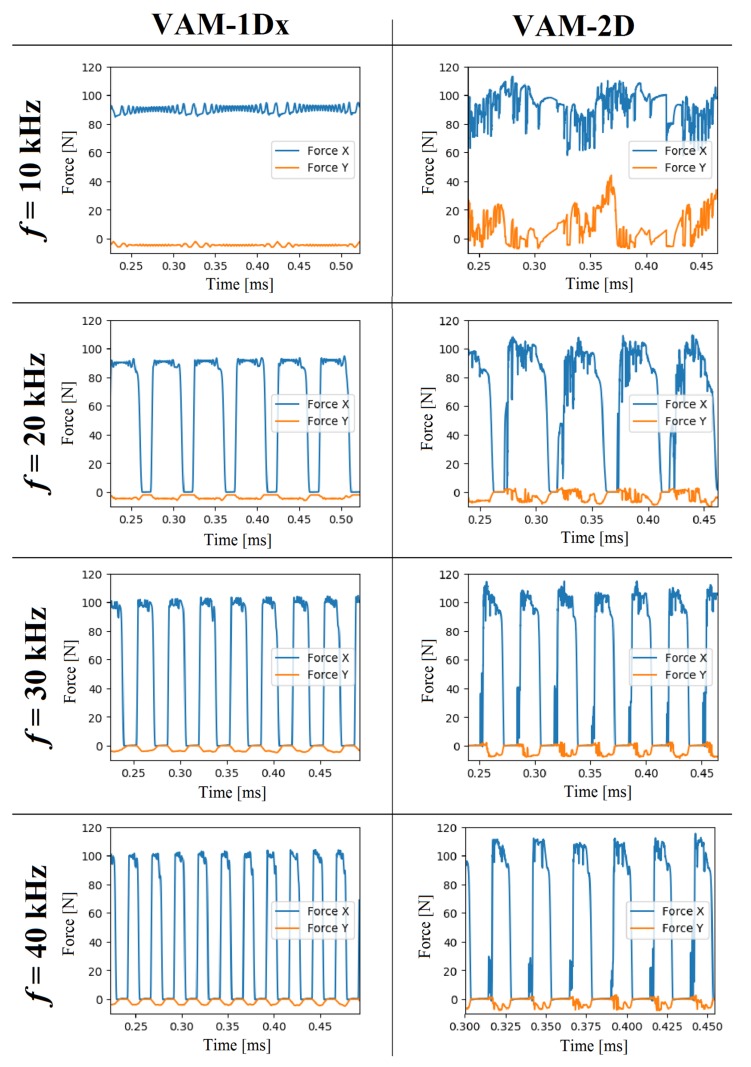
Cutting forces estimated in VAM-1Dx (first column) and VAM-2D (second column) when using vibration frequencies of 10, 20, 30, and 40 kHz.

Figure 5 exhibits cutting forces for two vibration-assisted cutting modes. The first column corresponds to the cutting forces of the tool vibrating in the horizontal direction mode (VAM-1Dx). The second column corresponds to the cutting forces for elliptical vibration of the cutting tool (VAM-2D). Looking at the cutting forces when using different vibration frequencies, we see that, at 10 kHz, the average cutting force was found between 88 and 91 N, and the maximum peak of the vibration cutting force reached values of 85 and 94 N. These maximum and minimum cutting forces appeared when the tool moved in the opposite and the same direction to the cutting feed, respectively. In this case, these maximum and minimum values of the cutting forces indicate that the tool and material were in contact during the entire vibration-assisted process. One can also note that the force values in the vertical direction showed negative values of force, due to the rake angle inclination of 6°. Additionally, the cutting forces in VAM-2D for frequencies of 10, 20, 30, and 40 kHz show that the peak values occurred when the tool moved in the direction opposite to the cutting feed, and the valleys occurred when the directions of the movement of the tool and the material were the same. VAM-2D combined with 10 kHz presented average forces in the range of 78 N to 102 N, in the horizontal component. The cutting forces in VAM-2D for vibration frequencies of 20, 30, and 40 kHz show that the horizontal component had a vibration behavior with no moment of force. Vertical forces showed negative values, due to the rake angle inclination of 6°. The horizontal component of the cutting force exhibited greater values than the vertical component, necessary to remove the chip.

**Figure 6:**
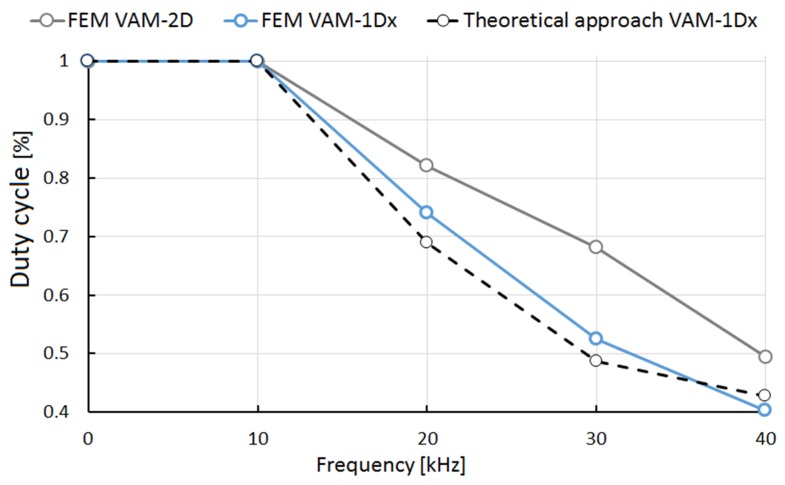
Duty cycle behavior for the different vibration frequencies used in VAM-1Dx and VAM-2D.

Some studies that showed the behavior of the cutting force in VAM presented an oscillatory tendency of the force in VAM-1Dx [41] and VAM-2D [42], similar to that presented in this study. These works also showed the thrust force, Fy, with values smaller than those of the shear force, Fx. Also, the residual stress on the surface of the VAM-machined material, observed in Figure 4, shows a behavior response similar to that in Naresh’s work [43]. However, no articles were found that showed the SCE in vibration-assisted machining. The conditions of cutting speed, cutting feed, and depth of cut are explicitly incorporated into the SCE, which makes this a more robust indicator of the cutting phenomenon. The lack of continuous contact during the VAM was determined by finding the moment when the cutting force and shear strain rate are equal to zero. Accordingly, Figure 6 shows the percentage of tool-material contact time for the different VAM conditions. Note that an increase in frequency decreased the contact of tool and workpiece; this situation influenced the cutting power consumption. The tool-workpiece contact ratios presented here in VAM-1Dx are similar to the theoretical values reported in Reference [14]. Also, it was evident that the contact ratio in VAM-2D was higher than the contact ratio in VAM-1Dx.

**Figure 7:**
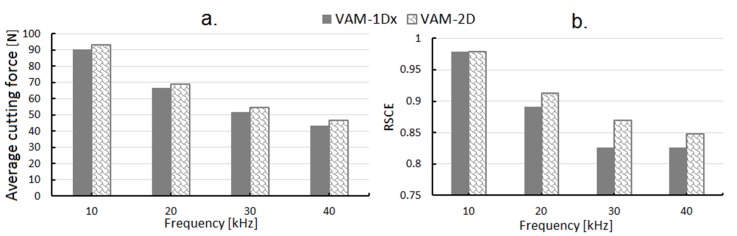
Average cutting force (**a**) and relative specific cutting energy (RSCE) (**b**) for frequencies of 10, 20, 30, and 40 kHz when using VAM-1Dx and VAM-2D.

Figure 7a shows the average cutting force and SCE for vibration frequencies of 10, 20, 30, and 40 kHz. The average force decreased when the frequency increased, due to the fact that the tool-material contact time decreased. The maximum force did not increase in the same proportion. In VAM-1Dx, the average cutting force decreased when higher frequencies were used. For a frequency of 20 kHz, no significant changes were observed in the average cutting force. Similarly, for higher vibration frequencies used in VAM-2D, smaller average cutting forces were found. 

On the other hand, SCE values were determined by using Equation (4), where the cutting power and the rate of material removal are estimated, as previously mentioned. Note that the cutting power and the rate of material removal were not constant values in the VAM conditions studied. For that reason, the average time frame of each of these magnitudes was used for the two vibration conditions, VAM 1Dx and 2D. Furthermore, the output speed was estimated using the same procedure as in the stationary orthogonal cutting. A line was drawn (see Figure 3b) and observed at the initial and final step times of the chip formation (undeformed chip thickness and deformed chip thickness respectively). The average output speed of the chip was determined using Equation (5), in order to then determine the average value of SCE for a vibration mode. Then, Equation (6) was used to compare the relative specific cutting energy (RSCE) between stationary and VAM orthogonal cutting processes.

(6) $$RSCE=\frac{SCE_{VAM}}{SCE_{stationary}}$$

Figure 7b shows RSCE for VAM frequencies of 10, 20, 30, and 40 kHz under constant cutting conditions of cutting feed, cutting speed, and depth of cut. Since the RSCE is a comparative value with respect to the stationary process, if values are lower than one, the energy required per unit volume of material removed in VAM is lower than that in a conventional turning process (without vibration). For a VAM of 10 kHz, the RSCEs of VAM-1Dx and VAM-2D were similar and slightly less than one. For VAM frequencies of 20, 30, and 40 kHz, frequencies that showed a lack of continuous contact between tool and workpiece, RSCE decreased in both vibration modes. In general, VAM-1Dx presented lower RSCE than VAM-2D, except for at 10 kHz where similar values of RSCE were observed. For the higher frequencies (30 and 40 kHz), RSCE seemed to stabilize at a constant value in VAM-1Dx. The stationary cut had an RSCE of one, and the VAM-1Dx cut, at a frequency of 30 kHz, had an RSCE of 0.82, evidencing a reduction of 0.18. These lower values of RSCE in the different VAM conditions can be associated with the intermittent contact between tool and workpiece. A lower value of the contact ratio is associated with a lower average of the cutting power. On the other hand, the shorter tool-material contact time can promote larger values for the maximum cutting force, a situation that would increase the average cutting power. Moreover, this lack of contact between the cutting tool and workpiece also leads to the absence of material removed during a short period of time, reducing the material removal rate and, thus, increasing the SCE. Therefore, the sum of all these behaviors, some of them antagonistic, makes VAM a promising cutting process which needs further investigation, as thoroughly described by Brehl et al. [14] and Wei-Xing et al. [44].

## 4. Conclusions

In the present work, an FEM approach was successfully developed and validated for orthogonal cutting, to study the multi-directional vibration-assisted cutting process in S235 carbon steel. Thanks to this, several cutting parameters were analyzed, such as the duty cycle, the shear forces, and the SCE, in the different vibration-assisted machining modes (VAM-1Dx, VAM-1Dy, and VAM-2D). Accordingly, the main findings are summarized below.

1. For the different VAM conditions, there were vibration frequencies where the cutting tool was not permanently in contact with the material to be cut. When this occurred, a lower average of cutting forces was observed.
2. The stationary orthogonal cutting presented a greater SCE than any of the studied VAM conditions (VAM-1Dx and VAM-2D). Therefore, VAM seems to be a more efficient process for cutting S235 carbon steel. This process means that the machinability improves with VAM.
3. In VAM-1Dy, the estimated cutting forces exhibited unreliable results, due to lack of conservation of energy. Moreover, the chip thickness displayed irregularities in the material surface in contact with the flank and rake faces of the cutting tool.
4. The behavior of SCE presented lower values for higher vibration frequencies, despite the fact that higher vibration frequencies lead to lower material removal rates. That is because cutting power decreased faster than the material-removal rate values, due to the intermittent contact between the cutting tool and the material to be cut. Accordingly, the material’s machinability was improved up to 18% when a VAM-1Dx with a vibration frequency of 30 kHz was used.

## Figures and Tables

**Table 1:** Vibration parameters for different vibration-assisted machining (VAM) modes. 1D—one direction; 2D—two directions.

Cutting Mode	Frequency (kHz)	Amplitude (µm)	
Stationary	0	0	0
VAM-1Dx	10	5	0
	20		
	30		
	40		
VAM-1Dy	10	0	5
VAM-2D	10	5	5
	20		
	30		
	40		

**Table 2:** Operational turning parameters and mechanical properties of S235 carbon steel.

Material	Steel S235
$C_{p}$ (J/kg·°C)	470
E (GPa)	190
α (°C^−1^)	12 × 10^−6^
D (kg/m^3^)	7800
Hardness (HRB)	62.8 ± 1.5
Spindle speed (rpm)	600; 900
Cutting feed (mm/rev)	0.07; 0.14
Depth of cut (mm)	0.2; 0.4

**Table 3:** Johnson–Cook parameters to define the mechanical behavior of S235 carbon steel.

A (MPa)	B (MPa)	C	n	m	$\overset{´}{\varepsilon_{0}}\;(s^{-1})$	$T_{room}\;({}^{\circ}C)$	$T_{melt}\;({}^{\circ}C)$
275	350	0.022	0.36	0.81	5.6 × 10^−4^	22	1537.85

**Table 4:** Experimental and numerical simulation of the cutting parameters in a stationary orthogonal cutting process.

Stationary	*V_c_* (m/min)	*f* (m/rev)	*a_0_* (mm)	ξ		*φ* (°)		*SCE* (W·s/mm^3^)	
				Exp.	FEM	Exp.	FEM	Exp.	FEM
Case 1	57.46	0.07	0.2	3.69	3.09	10.5	21.3	6.7	4.6
Case 2	57.46	0.14	0.2	2.96	2.58	10.7	21.4	4.4	3.1
Case 3	57.46	0.07	0.4	4.09	3.17	14.1	23.7	4.8	4.6
Case 4	57.46	0.14	0.4	3.06	2.46	18.9	24.8	3.4	4.1
Case 5	38.21	0.07	0.2	6.36	3.24	10.4	21.5	6.0	5.1
Case 6	38.21	0.14	0.2	4.32	2.76	10.8	21.5	3.6	4.8
Case 7	38.21	0.07	0.4	5.25	3.20	10.9	21.8	5.4	4.9
Case 8	38.21	0.14	0.4	3.34	2.62	17.2	24.2	4.2	3.7

## Nomenclature

**Parameter**	**Definition**	**Units**
*a*	Chip thickness	mm
*a_0_*	Undeformed chip thickness	mm
*A*	Initial yield stress	MPa
*A_X_*	Amplitude of vibration in x-axis	μm
*A_Y_*	Amplitude of vibration in y-axis	μm
*α*	Main shear zone	-
*α_T_*	Coefficient of thermal deformation	°C^−1^
*b*	Shear band thickness	mm
*B*	Hardening constant	MPa
*β*	Angular mismatch	Rad
*C*	Strain rate coefficient	-
*C_P_*	Specific heat	J/kg·°C
*D*	Density	kg/m^3^
*E*	Elastic modulus	GPa
$\overset{´}{\varepsilon }$	Strain rate	s^−1^
${\overset{´}{\varepsilon }}_{0}$	Initial strain rate	s^−1^
*f*	Cutting feed	mm/rev
*f_0_*	Vibration frequency	kHz
*F_C_*	Cutting force	N
ξ	Compression ratio	-
*L_i_*	Length of segment	mm
*m*	Coefficient of thermal softening	-
*n*	Coefficient of strain hardening	-
*N_C_*	Cutting power consumption	W
*QW*	Material removal rate	mm^3^/s
*RSCE*	Relative specific cutting energy	-
*SCE*	Specific cutting energy	W∙s/mm^3^
*T_melt_*	Melting temperature	°C
*T_room_*	Room temperature	°C
*Vc*	Cutting speed	m/min
*V_i_*	Normal component of the speed of each segment	mm/s
*Vo*	Output chip velocity	m/min
ϕ	Shear plane angle	°
